# Orbital myositis induced by botulinum toxin injection: A case report

**DOI:** 10.1111/srt.70021

**Published:** 2024-08-21

**Authors:** Joe Khodeir, Paul Ohanian

**Affiliations:** ^1^ Department of Dermatology Faculty of Medicine and Medical Sciences, University of Balamand, Saint Georges Hospital University Medical Center Beirut Lebanon; ^2^ Department of Family Medicine Faculty of Medicine and Medical Sciences, University of Balamand, Saint Georges Hospital University Medical Center Beirut Lebanon

Dear Editor,

This report describes a case of orbital myositis occurring 3 weeks after botulinum toxin treatment for periorbital wrinkles in a healthy male.

A 50‐year‐old healthy male presented with acute, severe right eyelid swelling and orbital discomfort 3 weeks after receiving a botulinum toxin (onabotulinumtoxinA) injection for periorbital and glabellar wrinkles. The patient denied any recent medication use or trauma to the area. Examination revealed bilateral eyelid swelling, more pronounced on the right side, with conjunctival hyperemia and painful diplopia (Figure 1A,B). Suspecting orbital cellulitis, the patient was initially treated with oral clindamycin and ciprofloxacin, but the edema worsened after 1 week (Figure 1C). He was then referred to an ophthalmologist, and an orbital MRI showed bilateral edema and increased signal intensity of the ocular muscles, more prominent on the right side, with enhancement and thickening of the inflamed extraocular muscles (inferior, medial, and inferior recti) and mild right orbital proptosis (Figure 2), leading to a diagnosis of orbital myositis. Comprehensive laboratory tests, including complete blood count (CBC), liver and kidney function tests, thyroid function tests, antinuclear antibodies, thyroid antibodies, IgG4 serum level, ANCA antibodies, erythrocytes sedimentation rate  (ESR), C‐reactive protein (CRP), serum angiotensin‐coverting enzyme (ACE), rheumatoid factor, herpes family polymerase chain reaction (PCR), echocardiography, and thorax and abdomen scan, were all negative. The patient was started on intravenous methylprednisolone (1 mg/kg) for 7 days, followed by a gradual tapering of oral prednisone over 6 weeks. Marked improvement was noted 5 days after initiating IV therapy, and total remission was observed after 2 weeks, with no relapse at the 8‐month follow‐up. During follow‐up, the patient mentioned experiencing very mild eyelid swelling after a botulinum toxin injection 4 years ago, which resolved in 2 weeks without intervention.

**FIGURE 1:**
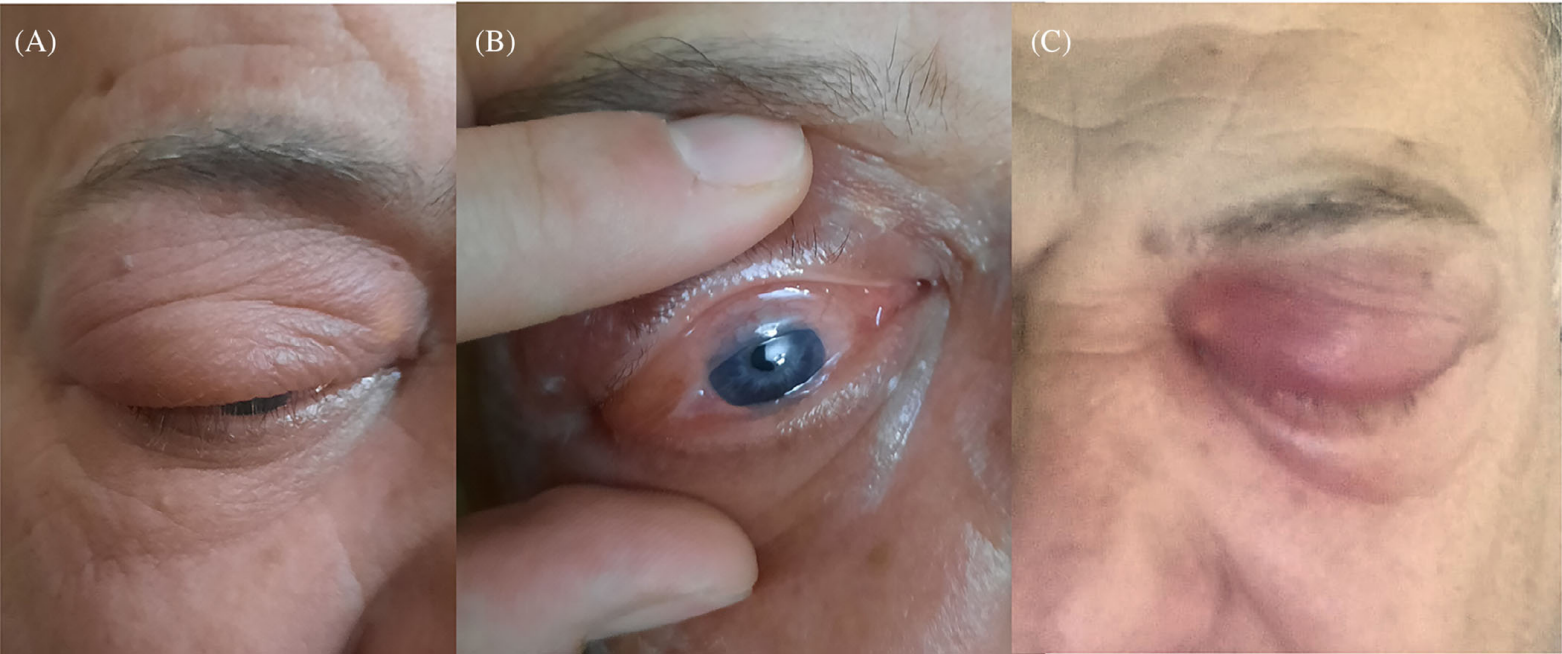
(A, B) Right eyelid swelling, conjunctival hyperemia, and chemosis. (C) severe right‐sided periorbital eyelid swelling limiting the patient's sight, after the failure of oral antibiotics.

**FIGURE 2:**
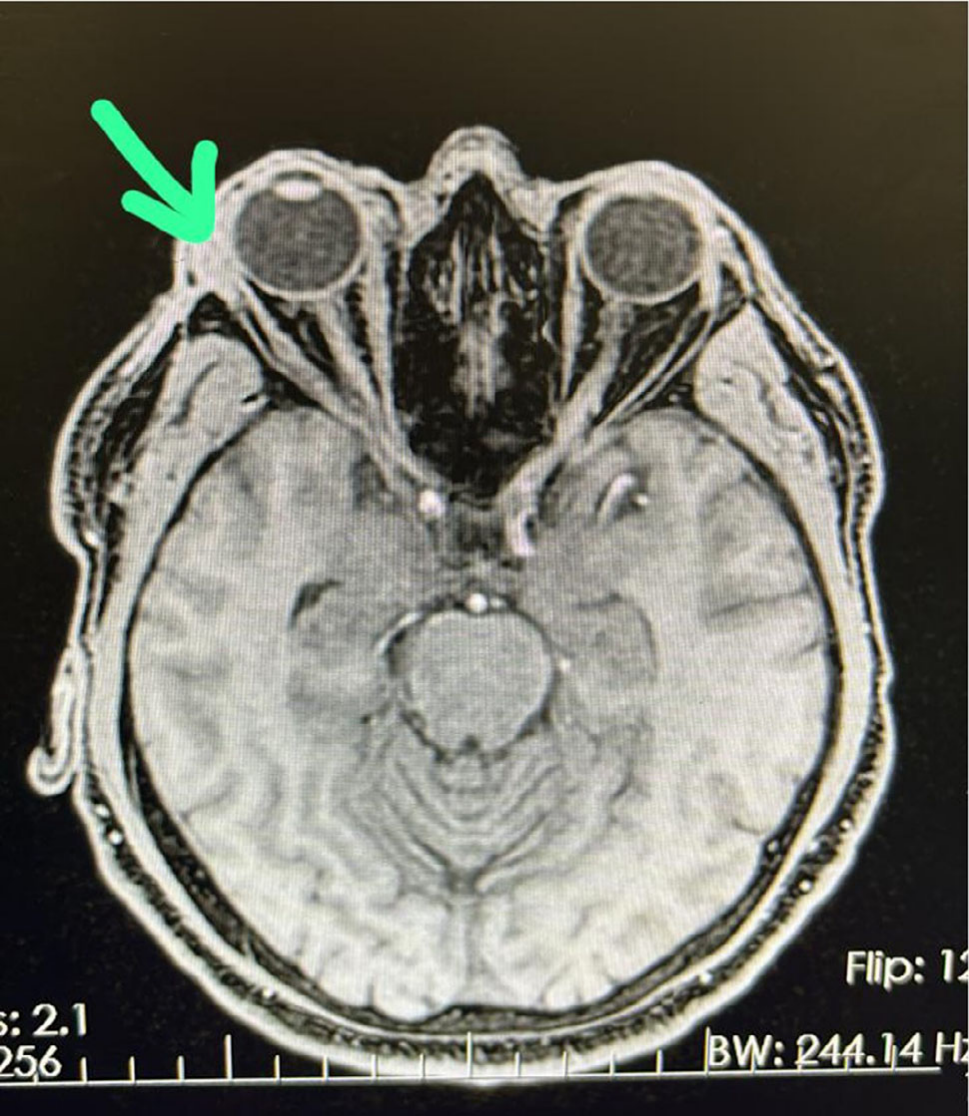
Orbital MRI showing bilateral edema and increased signal intensity of the ocular muscles, more prominent on the right side, with enhancement and thickening of the inflamed extraocular muscles (green arrow) and mild right orbital proptosis.

Orbital myositis is an inflammatory condition of the extraocular muscles, typically associated with autoimmune diseases, infections, and neoplastic conditions.^1^ It often presents acutely with symptoms such as eyelid swelling, orbital pain, and diplopia.^2^ In this case, the patient developed symptoms 3 weeks after a botulinum toxin injection, which is not a commonly reported trigger for orbital myositis. To our knowledge, no cases of orbital myositis associated with botulinum toxin injections have been reported, making this the first documented case. The diagnosis of orbital myositis in this patient was supported by clinical presentation, MRI findings, and the exclusion of other potential secondary causes. Comprehensive laboratory tests and imaging ruled out autoimmune disorders, infections, and neoplastic conditions. The temporal relationship between the botulinum toxin injection and the onset of symptoms, coupled with the patient's history of a similar but milder reaction to a previous botulinum toxin injection, strongly suggests a causal link rather than an idiopathic case.

The pathophysiology linking botulinum toxin to orbital myositis might be related to an inflammatory response induced by the toxin.^1^, ^3^, ^4^ A study by Pingel et al. demonstrated that intramuscular botulinum toxin injections cause a systemic inflammatory response and a local inflammatory response in muscle tissue of rats, with significantly elevated cytokine levels observed up to 4 weeks postinjection.^3^ This inflammatory response could lead to the development of orbital myositis in susceptible individuals following botulinum toxin injections, as observed in our patient. Clinicians should be aware of this rare but serious adverse effect and consider it in differential diagnoses of postinjection ocular symptoms.

## CONFLICT OF INTEREST STATEMENT

The authors declare no conflict of interest.

## ETHICS STATEMENT

Written informed consent was taken from the patient to publish the figures.

## Data Availability

The data used to support the findings of this study are included in the article.
